# In Vivo Degradation Studies of PGA-PLA Block Copolymer and Their Histochemical Analysis for Spinal-Fixing Application

**DOI:** 10.3390/polym14163322

**Published:** 2022-08-16

**Authors:** Seung-Kyun Yoon, Dong-June Chung

**Affiliations:** School of Chemical Engineering, SungKyunKwan University, 2066 Seobu-Ro, Jangan-Gu, Suwon-Si 16419, Gyeonggi-Do, Korea

**Keywords:** PGA–PLA block copolymer, bio-resorbable, in vivo degradation test, histochemical analysis

## Abstract

Polylactic acid (PLA) and polyglycolic acid (PGA) are well-known medical-implant materials. Under the consideration of the limitations of degradable polymeric materials, such as weak mechanical strength and by-product release through the biodegradation process under in vivo environments, PLA–PGA block copolymer is one of the effective alternative implant materials in the clinical field. In our previous study, two types of extremely effective PGA–PLA copolymers (multi/tri-block PGA–PLA copolymers) were synthesized. These synthesized block copolymers could overcome aforementioned issues and also showed good biocompatibility. In this study, the PGA–PLA block copolymers with large molecular weight were synthesized under the same chemical scheme, and their bio durability was confirmed through the in vivo degradation behavior and histochemical analyses (by hematoxylin and eosin and immune staining) in comparison with commercial PLGA random copolymer (medical grade). Specimens for the degradation test were investigated by SEM and X-ray diffractometer (XRD). As a result, the synthesized PGA–PLA block copolymer showed good biocompatibility and had a controlled biodegrading rate, making it suitable for use in resorbable spinal-fixation materials.

## 1. Introduction

In the orthopedic surgery for vertebral disease therapy, the need for resorbable materials for implants was steadily increased for the treatment of older patients. The use of resorbable polymeric implanting materials can lessen the difficulty for the patient, such as secondary surgeries for removing the implanted device. Among the resorbable materials for medical use, polylactic acid (PLA), polyglycolic acid (PGA), and their random copolymer (PLGA) were consistently researched, developed, and widely used for clinical applications [1,2,3]. Commercial random PLGAs must, however, overcome their inadequate mechanical strength and fast-degrading characteristics under in vivo condition of load-bearing applications within 3–6 months if they are to be used in the orthopedics field [4,5]. In our previous study [6], PGA–PLA block copolymers (inherent viscosity: 0.9486 dL/g (30 °C, 0.5 g/dL, Hexafluoroisopropanol (HFIP))) that had a longer degradation period than random PLGA were synthesized and reinforced by blending with hydroxyapatite-grafted PLA and PGA fiber to improve their mechanical property (for example, tensile strength of more than 100 MPa). Synthesized copolymers demonstrated good biocompatibility through in vitro and in vivo biosafety studies, as well as improved mechanical properties through reinforcing processes. However, synthesized block copolymers (with/without reinforcing) showed complete biodegrading behavior 6 months after implantation and were found to be insufficient for long-term (more than 6 months) implantation [6]

One of the reasons for the complete degradation of block copolymers over 6 months was that the residual moisture in 1,4-butanediol (used as initiator in the synthesis of PLA block in synthetic scheme, moisture content: 500–2000 ppm) suppress the polymerization rate and molecular-weight increment [7,8].

In this study, to improve the molecular weight of PGA–PLA block copolymer, anhydrous ethylene glycol (residual water content: less than 100 ppm, as alternated initiator for 1,4-butanediol) was used to synthesize PLA block (PLA-diol) and then this PLA-diol was used as the starting moiety to synthesize high-molecular-weight PGA–PLA block copolymer. The molecular-weight increment of synthesized PGA–PLA block copolymer was confirmed by inherent viscosity, glass transition, and melting-temperature (Tg and Tm) measurement. For the investigation of sustained degradation behavior, the obtained PGA–PLA block copolymers were conducted by an in vivo biodegradation test covering 6 months with random PLGA as the control. Generally, the degradation characteristics under in vivo conditions were affected by surface morphology, crystallinity, the molecular weight of the polymer, and so on [9]. According to the predetermined schedule, the specimens for the in vivo degradation test using SD-rats were investigated using SEM and TGA to confirm the surface erosion and thermal stability of block copolymers. The specimens for the initial stage of the in vivo degradation test covering 3 months were examined by X-ray diffractometer (XRD) measurement, specifically to demonstrate the sustained degradation tendency of the PGA–PLA block copolymer backbone. Additionally, paraffin-embedded samples, including the tissues surrounding the SD-rat specimen-insertion site, were examined by histochemical analysis for the confirmation of a foreign-body reaction during the period of deterioration. From these results, the synthesized PGA–PLA block copolymers showed enhancing biocompatibility, mechanical strength (over 120 MPa in flexure strength), and sustained biodegradation behaviors for a long period compared with a random PLGA copolymer and, therefore, were shown to be suitable materials for long-term implantation (more than six months).

Furthermore, the above-mentioned improved mechanical strength is expected to enhance the mechanical properties through combination with a reinforcing agent (such as PLA fiber) in the next step.

## 2. Materials and Methods

### 2.1. Materials

L-Lactide, glycolide (LA, GL, >99% purity, respectively), and tin (II) 2-ethylhexanoate (Sn(Oct)2), as the monomers and catalyst for ring-opening polymerization, were purchased from Medichem (Gongju, Korea) and Sigma-Aldrich Co., Ltd. (St. Louis, MO, USA), respectively. Anhydrous ethylene glycol, as an initiator for the ring-opening polymerization, was purchased from Sigma-Aldrich Co., Ltd. (St. Louis, MO, USA). Random poly (lactide-co-glycolide) (PLGA, PLA: PGA = 30:70 mole ratio, inherent viscosity: 1.0 dL/g (25 °C, in HFIP) was purchased from Meta Biomed Co., Ltd. (Cheongju, Korea).

### 2.2. Experimental Method

#### 2.2.1. Synthesis PGA–PLA Block Copolymer

**Figure 1:**
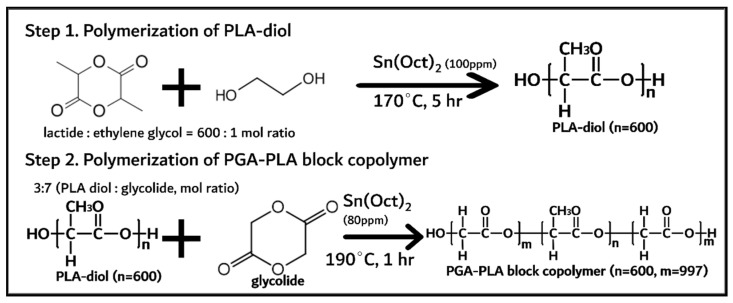
Synthesis of PGA–PLA block copolymer.

PGA–PLA block copolymer was synthesized in two steps and shown in Figure 1.

PLA diol was first synthesized via the ring-opening polymerization of lactide (144 g, 1 mol) with initiator and catalyst (anhydrous ethylene glycol (0.24 g, 1.6 mmol) and Sn(Oct)_2_ (100 ppm) as initiator and catalyst, respectively) at 170 °C for 5 h under a nitrogen atmosphere [10]. The monomer (lactide), initiator, and catalyst were quantified and vacuum-dried for 1 h before polymerization. The obtained PLA diol was treated under vacuum conditions (<1 torr) at 120 °C for 4 h to remove any unreacted monomers (lactide < 0.5%). Next, the PGA–PLA block copolymer was synthesized via the ring-opening polymerization of glycolide (61 g, 0.52 mol) with the presynthesized PLA diol (32 g, 0.22 mol) at 190 °C for 1 h under a nitrogen atmosphere. The obtained PGA–PLA block copolymer was treated under the same conditions (vacuum-dried for 4 h) to remove any unreacted monomer (<0.5%).

The synthesized PGA–PLA block copolymer was characterized via thermal analysis using differential scanning calorimetry (DSC, TA instruments Inc., DSC Q200, New Castle, DE, USA, heating rate: 10 °C/min, temperature range: 0~240 °C under nitrogen) and using gel-permeation chromatography (GPC, elute of HFIP and 0.01 N sodium trifluoroacetate (NaTFA), flux rate; 0.3 mL/min, concentration: 3 mg/mL, PMMA standard condition, Tosoh bioscience, EcoSEC HLC-8320GPC, Tokyo, Japan at 40 °C) for molecular-weight measurement.

Block copolymer’s chain microstructure was examined using ^13^C nuclear magnetic resonance spectroscopy (^13^C-NMR, JEOL Ltd., JNM-ECZ500R, Tokyo, Japan) with trifluoroacetic acid-d (as solvent, Sigma-Aldrich Korea, Seoul, Korea) and universal testing machine (UTM, 2 mm/min, span: 64 mm, INSTRON^®^, INSTRON 3365, Norwood, MA, USA) according to ISO 178 (2019). Crystallinity of the synthesized block copolymer was also assessed by XRD, (Malvern Panalytical, AERIES 600, Worcestershire, UK) using Cu Kα radiation at a scanning speed of 5 °C/min.

#### 2.2.2. In Vivo Animal Degradation Test and Histochemical Analysis

**Figure 2:**
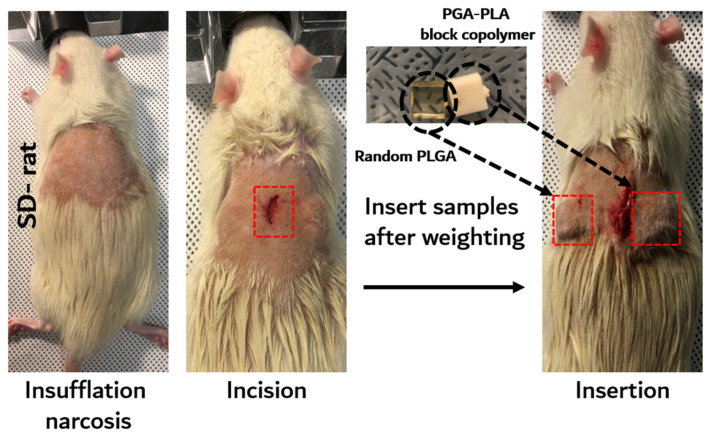
Scheme image of in vivo animal degradation test, which is sample insertion procedure.

The in vivo degradation test was reviewed and approved by the Institution Animal Care and Use Committee (IACUC) of Sungkyunkwan University, School of Medicine (SUSM) (Approval No. SKKUIACUC2019-08-21-1). 3Rs, Replacement of animals by alternatives wherever possible, Reduction in number of animals used and Refinement of experimental conditions and procedures to minimize the harm to animals were ensured in this in vivo degradation test. For investigating biodegradation behaviors, synthesized PGA–PLA block copolymers and commercial PLGA random copolymer (as a control group) were inserted in the Sprague Dawley rats’ (SD-rat, *n* = 3, 8 weeks old, Orient Bio Inc., Seongnam, Korea) intradermal back skin (Figure 2).

The PGA–PLA block copolymer and PLGA random copolymer cube-type implantation specimens (10 × 10 × 4 mm, length×width×height) for the in vivo test were produced using an injection-molding device (Mini Molder BA-915A, Bautek Co. Ltd., Pocheon, Korea) under processing temperatures between 220 °C and 240 °C and were sterilized by ethylene-oxide gas before implantation. Sample insertion was conducted under insufflation narcosis using isoflurane (0.5–2.0%) with O_2_ gas for the fixation process. After inserting the specimens, the incision site was sutured by nonabsorbable EZ clip wound closures (Stoelting Co., Wood Dale, IL, USA). Furthermore, 40 mL/kg of Metacam^®^ (Boehringer Ingelheim Co., Ingelheim am Rhein, Germany) was subcutaneously injected once per day for pain management.

The SD rats were subsequently sacrificed according to the predetermined time schedule (once a month up to half a year after implantation surgery) and inserted specimens were extracted for histochemical analyses with the surrounding tissue [6]. The residual tissues on the inserted PGA–PLA block copolymer and random PLGA copolymer specimens were gently removed from the harvested samples and dried in vacuo. Next, the weights of the harvested block and random copolymer specimens were measured (Adventurer™ AR2140, Ohaus Co., Pine Brook, NJ, USA) for the confirmation of biodegradation.

Surficial morphologies of specimens of 1 and 2 months after insertion were observed by scanning electron microscope (SEM, JSM7000F, Jeol Co., Ltd., Tokyo, Japan) for the investigation of surface erosion [8]. The crystallinity of the specimens c 1 for 3 months after insertion was assessed by X-ray diffractometer using Cu Kα radiation at a scanning speed of 5 °C/min.

The surrounding tissues of the PGA–PLA block copolymer specimens in the back sites of the SD rats were subjected to fixation procedures with paraffin for biopsy sample. Extracted tissues around the samples were fixed by 10% neutral buffered formalin (NBF, BN-019, Biosesang Co., Ltd., Seungnam, Korea). Fixation was performed after more than 2 days, and the NBF solution was refreshed every day. Next, fixed tissues were trimmed by razor and dehydrated by ethanol and xylene, respectively. The staining process for histochemical analyses was conducted using hematoxylin and eosin and CD-68 antibody (Thermo Fisher Scientific, Rockford, IL, USA) to detect the nuclei of the associated macrophages owing to foreign-body reaction. Digital images of the paraffin-embedded tissue sections were obtained using slide scanner scope (Aperio ScanScope^®^ CS system, Leica Biosystems, Wetzlar, Germany).

## 3. Results and Discussion

### 3.1. Synthesis of PGA–PLA Block Copolymer

Synthesized PLA moiety and PGA moiety in block copolymer were characterized by DSC, GPC, and flexural-strength measurement, and their data are summarized in Table 1.

**Figure 3:**
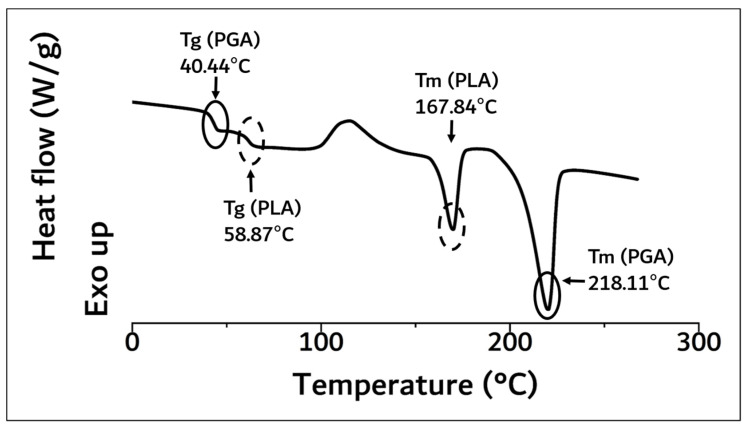
DSC curve of synthesized PGA–PLA block copolymer. (0 °C~270 °C, heating rate: 10 °C/min, 2nd run, N_2(g)_ condition).

As a result of DSC analysis in Figure 3, the Tg and Tm of the PLA moiety in block copolymer were 58.87 °C and 167.84 °C, and those of PGA were 40.44 °C and 218.11 °C, respectively.

**Figure 4:**
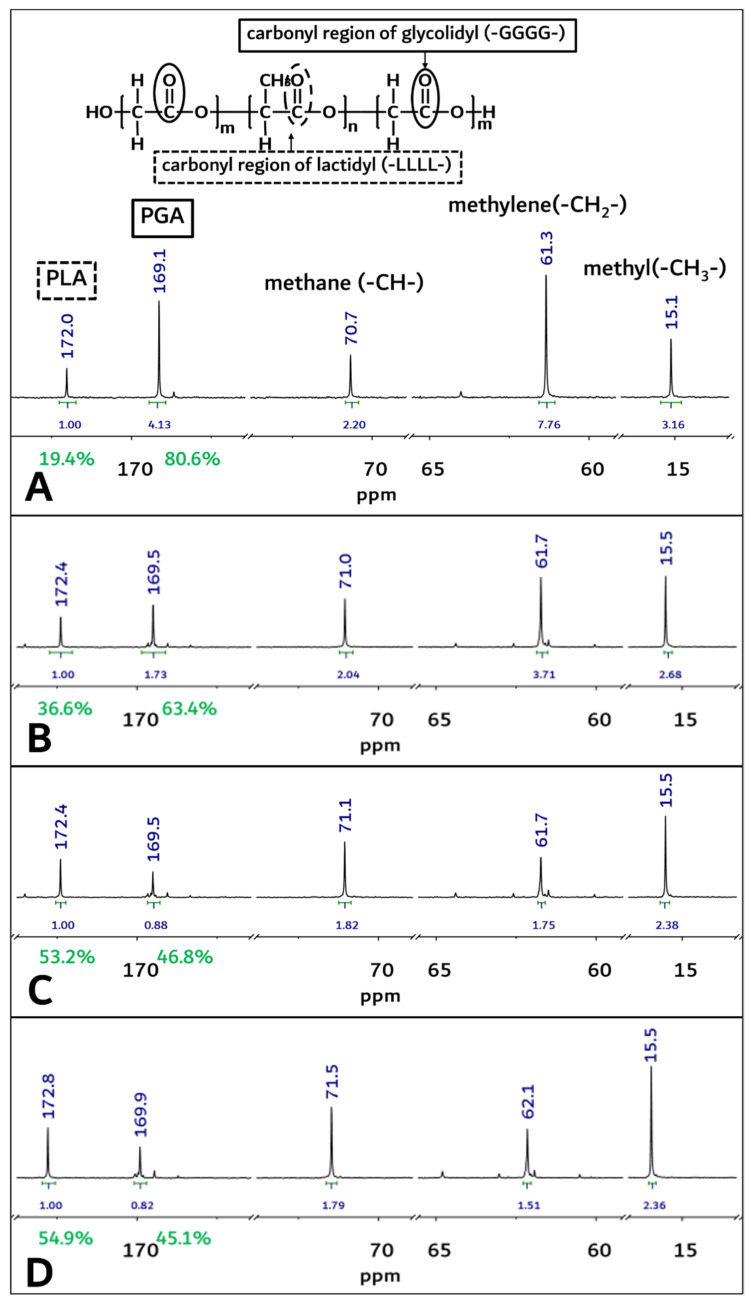
The ^13^C-NMR spectra of PGA–PLA block copolymer. (solvent: Trifluoroacetic acid-d, (**A**) Original sample’s spectra before implantation, (**B**) 1-month sample’s spectra after surgery, (**C**) 2-month sample’s spectra after surgery, (**D**) 3-month sample’s spectra after surgery).

These results were concurrent with recently reported papers [6,8,10,11,12]. The molecular weight of the polymerized PGA–PLA block copolymer was confirmed to be 159,000 (Mw) by GPC data analysis (PDI: 2.04, data not shown). Because of the molecular-weight increment in this study (inherent viscosity: 1.22 dL/g (25 °C, 0.5 g/dL, Hexafluoroisopropanol (HFIP))), the flexural strength of the newly synthesized PGA–PLA block copolymer was also increased to 137 MPa compared to relevant data in our previous report [6,10]. The copolymer’s mole-fraction ratio of PGA/PLA was determined by ^13^C-NMR spectroscopy and summarized in Figure 4.

From Figure 4A, homosequential-centered-integration-ratio data based on the carbonyl region of the glycolidyl (-GGGG- at 169.2 ppm) moiety and the lactidyl (-LLLL- at 172.2 ppm) moiety were obtained as 80.6: 19.4 (PGA/PLA mole ratio) in the block copolymer. However, these PGA and PLA mole ratios in the block copolymer were changed through an in vivo degradation test period, and the mole fraction of the PGA in the copolymer decreased from 80.6% to 45.1% at 3 months after insertion surgery (Figure 4A,D).

**Figure 5:**
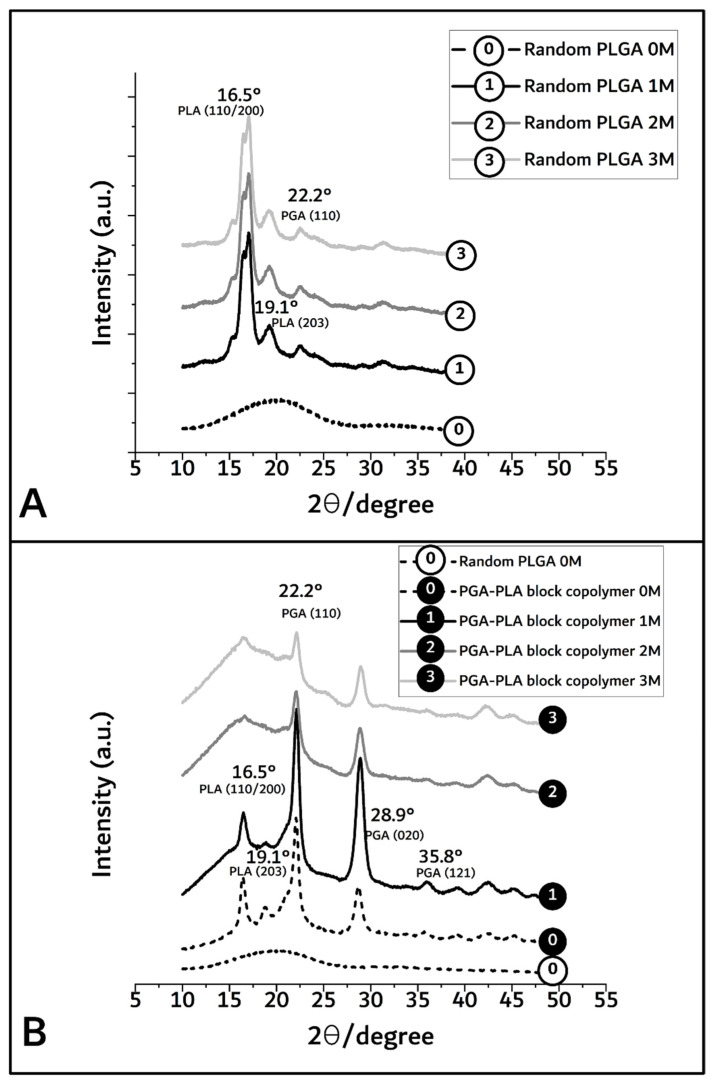
XRD spectra of random PLGA (**A**) and PGA–PLA block copolymer (**B**), harvested 0 to 3 months after insertion.

By XRD diffractometer (scanning speed; 5°/min, scanning region (2θ); 10° to 50°) measurement, the crystallinity differences owing to the biodegradation period of PGA–PLA block copolymer were shown to range from 92.2% (before insertion) to 63.1%, 53%, and 41% covering 1, 2, and 3 months after insertion (based on each peak area of Figure 5).

In Figure 5A,B, the broad peaks between 16° and 23° in the random PLGA polymer 0M (as control sample) indicate the amorphous region [13]. However, in the PGA–PLA block copolymer, semi-crystalline PLA block peak according to (I_110/200_) weakly appeared at 16.5°, and crystalline PGA block peaks according to (I_110_, I_020_) and (I_121_) are shown in 22.2°, 28.9°, and 35.8°, respectively [14,15].

In the case of random PLGA, the PGA moiety was rapidly degraded and quickly disappeared through its own crystalline characteristics over the course of the biodegradation time, but the semi-crystalline PLA moiety showed relatively slow degradation behavior and maintained its characteristics over the 3-month in vivo biodegradation test [16,17,18].

From the prementioned GPC, DSC, and XRD data of the synthesized PGA–PLA block copolymer, it was confirmed that a single backbone polymer including PLA and PGA blocks was synthesized according to the chemical scheme, as shown in Figure 1.

### 3.2. In vivo Degradation Test

**Figure 6:**
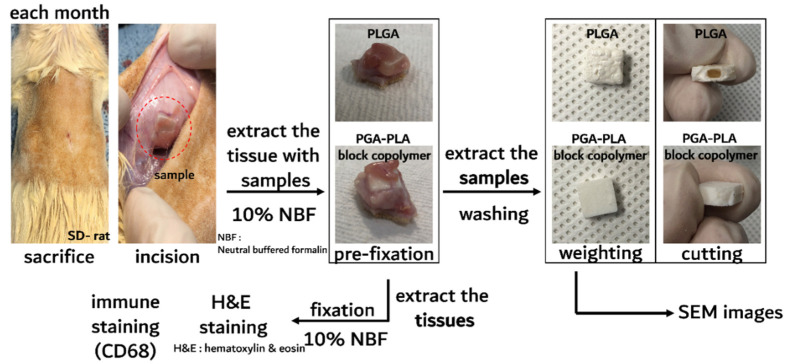
Schematic image of in vivo animal-degradation test showing sample-extraction procedure and samples after harvesting steps.

As shown in Figure 6, the specimens implanted in the intradermal back skins of the SD rats were removed according to the predetermined schedule.

**Figure 7:**
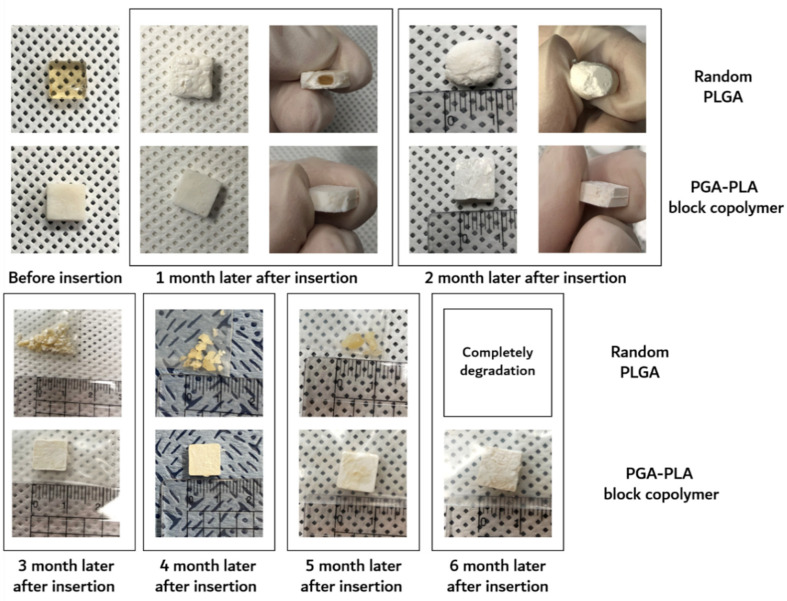
Image of the extracted in vivo animal-degradation-test samples (interval of extraction: 1 month, SD-rat, *n* = 3).

Furthermore, the surface morphological changes (including surficial and cross-sectional areas), weights of the dried specimens after their removal from the implanted site were investigated by SEM, digital camera, and electronic balance, respectively. Digital-camera images of the extracted specimens covering 6 months are summarized in Figure 7.

**Figure 8:**
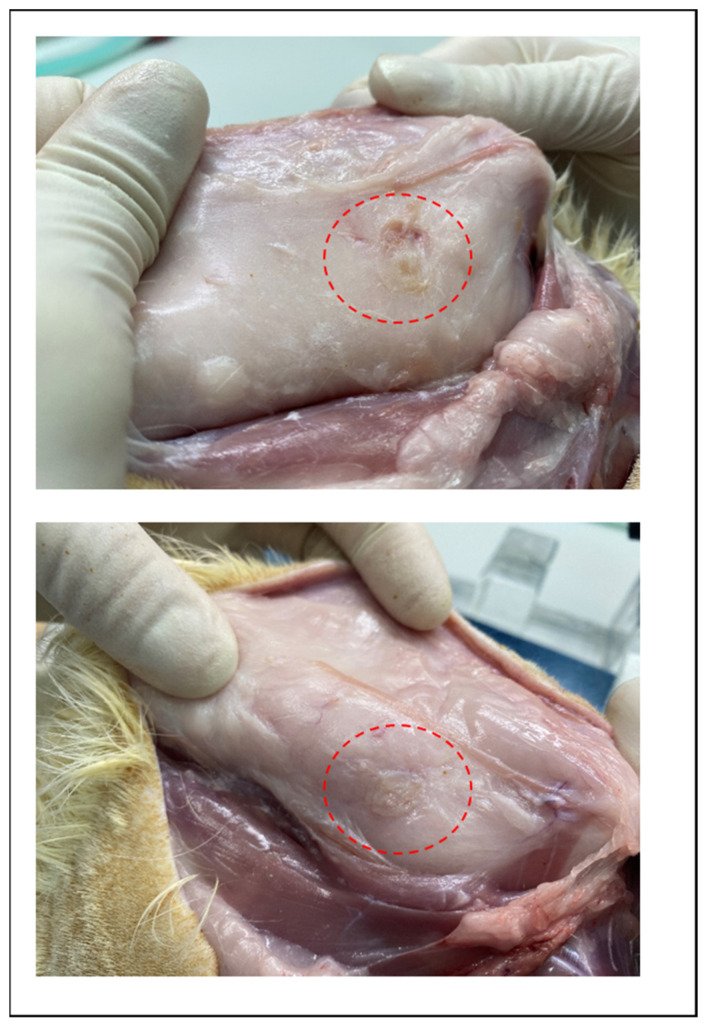
Images of incised back skin of SD rat which is degradation vestige of random PLGA in 6 months after insertion.

The degradation behaviors of the implanted copolymer specimens were investigated using weight changes. Generally, biodegradation occurred through surface hydrolytic erosion and sequential bulk degradation [9]. Surface and bulk erosions were affected by many parameters, such as the shape, crystallinity, porosity, and tortuosity of the polymeric materials [19]. From the SEM and optical-observation-analysis data, surface swelling was observed in all of the random PLGA samples up to 6 months. In particular, the surfaces of random PLGA specimens were found to be mashed in shape and crushed into small pieces 3 months after surgery. In random PLGA specimens 6 months after insertion, the implanted specimens were found to be vestiges in the incised back skins of the SD rats (Figure 8).

However, even 6 months after insertion, the surfaces of the PGA–PLA block copolymer specimens still exhibited their original morphologies and shapes. Therefore, the crystallinity alterations in the extracted samples were examined among the key reasons for this occurrence in order to validate such differences in biodegradation behavior between the random PLGA and block PGA–PLA copolymer. As shown in Figure 5A, amorphous peaks of random PLGA (0M) were observed at 16.5°, 19.1°, and 22.2° according to PLA (I_110/200_ and I_203_) and PGA (I_110_), as semi-crystalline peaks originating from homo PLA and PGA polymer [20,21,22]. Because the amorphous and crystalline regions of the random PLGA degraded at different rates, the relative proportion of the surviving crystalline sections increased as a result of progressive degradation in vivo [23].

Meanwhile, Figure 5B shows that the intensities of the PGA peaks at 22.2°, 28.9°, and 35.8° (PGA (I_110_, I_020_, and I_121_, respectively [14,15]) were decreased and that the PLA peaks at 16.5°, 19.1°, and 22.2° (PLA (I_110/200_ and I_203_)) were temporarily increased 1 month after insertion and remarkably decreased over 3 months. In the PGA–PLA block copolymer in the 1M-to-3M specimens, the intensity of the PGA peaks was decreased owing to the hydrolytic degradation of the crystalline parts. The PLA had a hardly swellable molecular structure compared with the PGA and PLA moieties in the block copolymer, hydrophobic methyl group in the semi-crystalline PLA block. By contrast, the PGA easily absorbed water. Consequently, it underwent hydrolysis and rapidly degraded.

Therefore, 3 months after surgery, the broad peak region was shown as random PLGA and PLA peaks, and the PGA peaks faded out due to degradation [16,17]. The greatest amount of crystallinity was found in the PGA peaks (22.2°, 28.9°, and 35.8°, 1M) and decreased by degradation (3M). However, the data for the crystalline PLA peaks (16.5° and 19.1°) up to 1 month revealed structural and morphological changes, including the formation of cleavage-induced crystallization and lamellar stacks, and disappeared after 2 months through lamellar-stack collapse [18].

**Figure 9:**
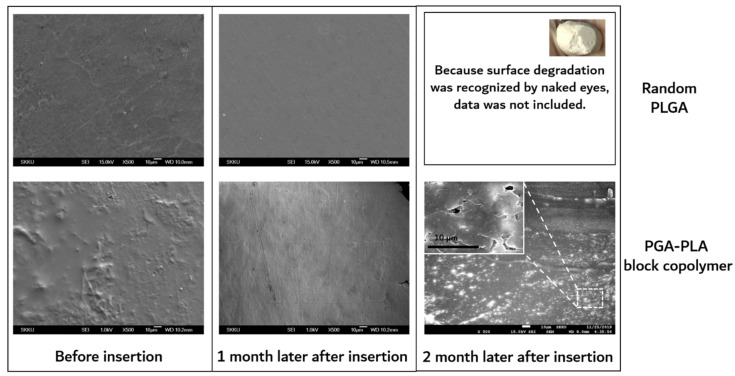
SEM images of surfaces of extracted samples, which were harvested after 0 to 2 months’ insertion.

As shown in Figure 9, the surfaces of the extracted block copolymer samples were investigated by SEM for the confirmation of surface erosion.

On the other hand, substantial surface and bulk erosion and complete degradation occurred in the random PLGA throughout the same implantation period. According to the previous report, the amorphous region of the polymer had a relatively easy path of water diffusion (the cause of the hydrolytic degradation) compared with the crystalline region of the polymer. The amorphous region contained disordered chain folds, chain ends, and tie-chain segments. At the interface between the amorphous and crystalline regions, hydrolysis occurred. This hydrolytic degradation led to the scission of the amorphous chain, causing a decrease in molecular weight and an increase in mobility [19].

**Figure 10:**
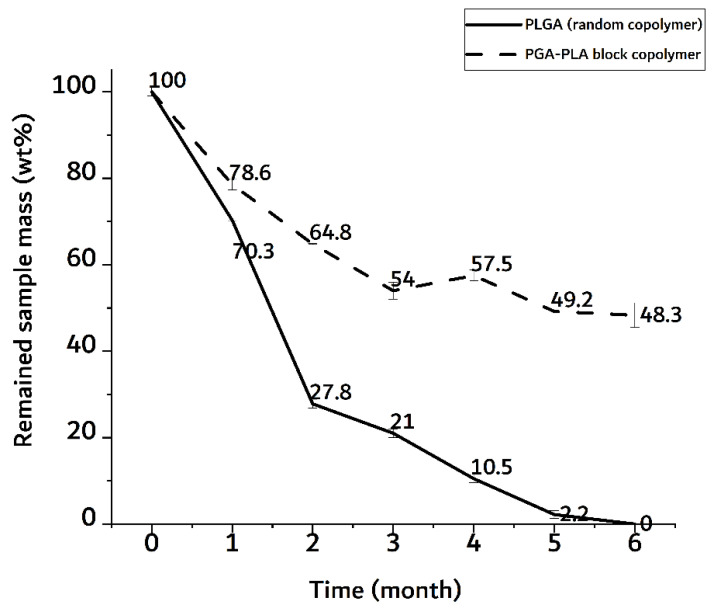
Weight trace of monthly extracted samples over 6 months after insertion surgery.

In Figure 10, the random PLGA sample showed a rapid weight decrease of up to 70 wt% in the 2-month implantation sample.

**Figure A1:**
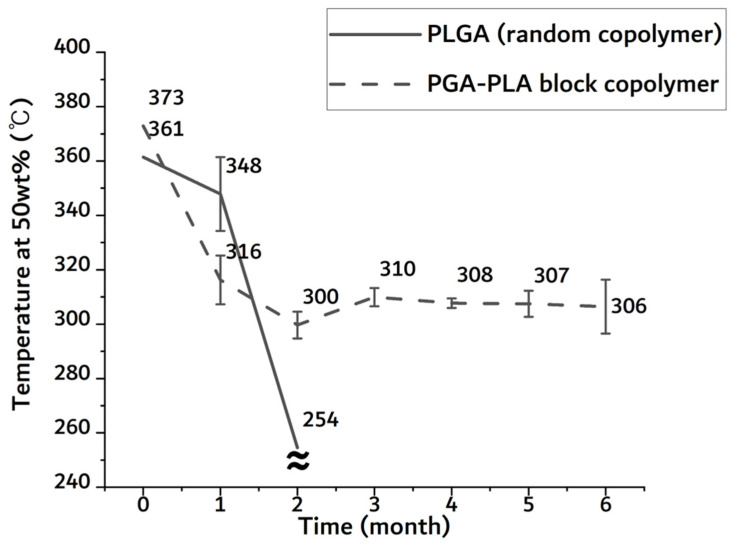
Temperature of half-mass trace of extracted sample 0 to 6 months after insertion from TGA.

The PGA–PLA block copolymer sample, on the other hand, showed a decrease in weight of up to 50 wt% in a 6-month implantation sample. In the random PLGA, their rapid decrease in thermal stability and their own weight were caused by the bulk degradation mentioned above [9]. And the meaningful differences time-depending degradation behavior of PGA-PLA block copolymer. (Appendix A, Figure A1)

**Figure 11:**
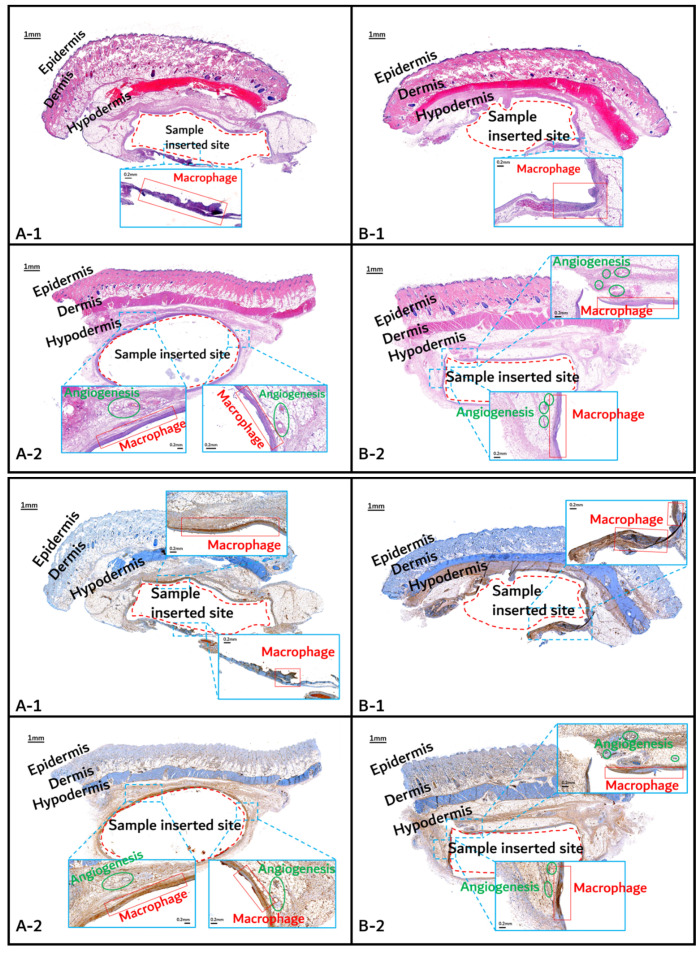
Histochemical analysis (upper: H&E staining, below: immunostaining) of in vivo animal degradation test. (**A-1**, **2**: tissue around a PLGA sample which 1 and 2 months, **B-1**, **2**: tissue around a PGA–PLA block copolymer sample that was inserted at 1 and 2 months).

**Figure 12:**
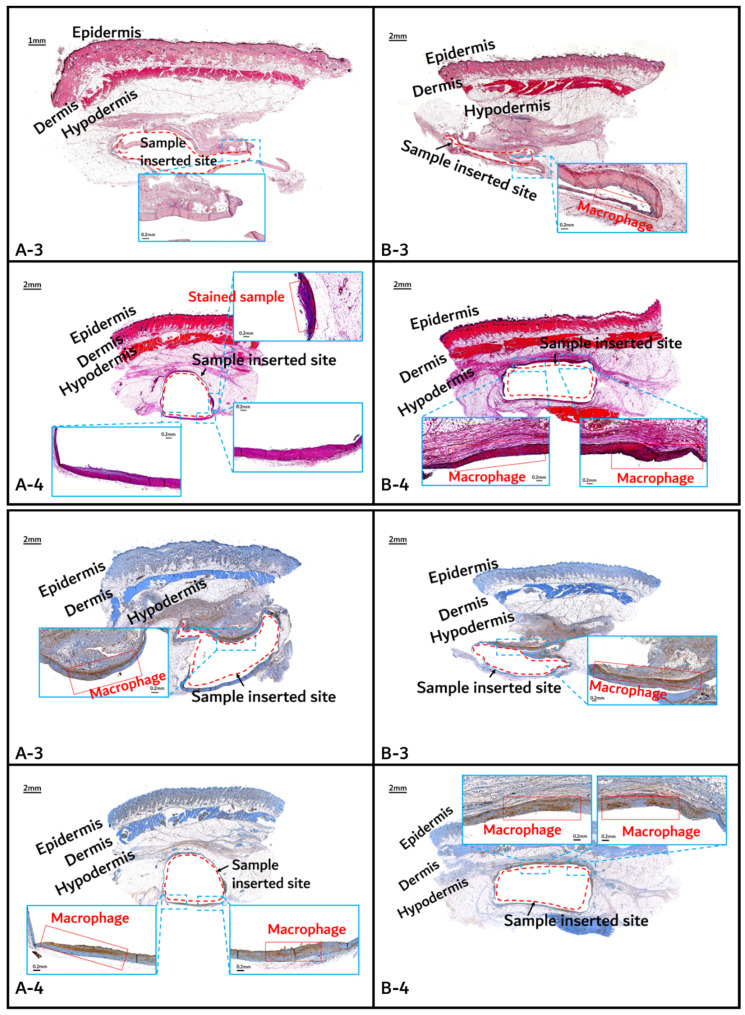
Histochemical analysis (upper: H&E staining, below: immunostaining) of in vivo animal degradation test. (**A-3**, **4**: tissue around a PLGA sample which 3 and 4 months after insertion, **B-3**, **4**: tissue around a PGA–PLA block copolymer sample that was inserted 3 and 4 months).

**Figure 13:**
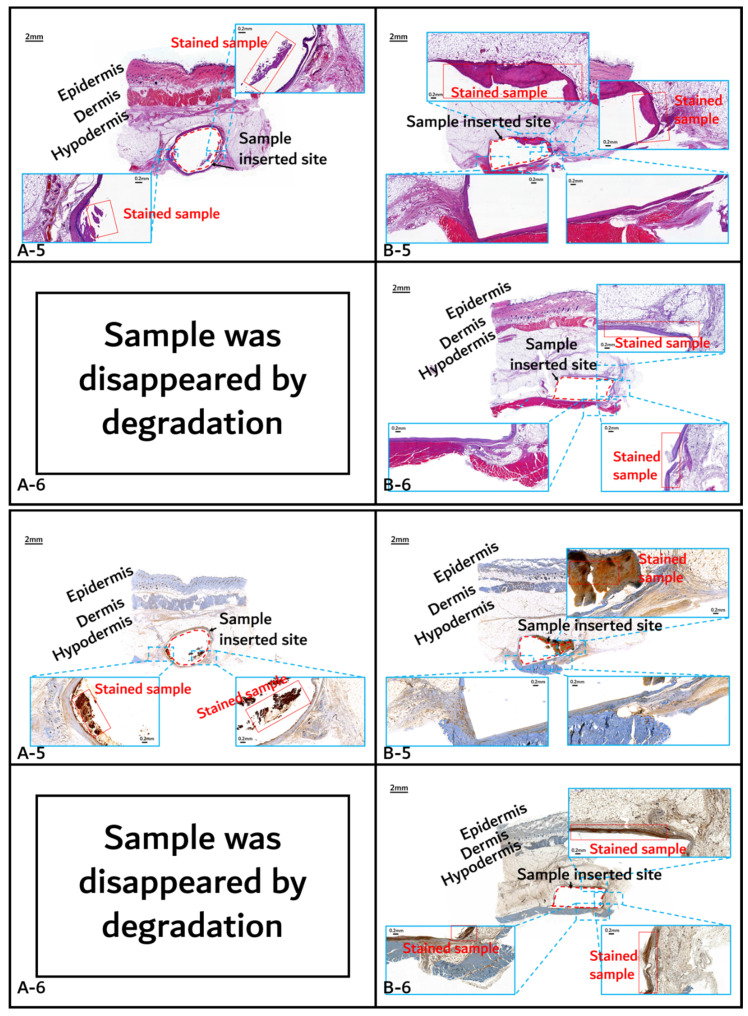
Histochemical analysis (upper: H&E staining, below: immunostaining) of in vivo animal degradation test. (**A-5**, **6**: tissue around a PLGA sample which 5 and 6 months after insertion, **B-5**, **6**: tissue around a PGA–PLA block copolymer sample that was inserted at 5 and 6 months).

The histochemical analysis results with H&E staining and CD-68 immunostaining are shown in Figure 11, Figure 12 and Figure 13.

Initial granulomatous inflammation and fibrous encapsulation were observed at an early stage of insertions up to 2 months (Figure 11). This phenomenon was closely related to rapid surface swelling (leading to surficial erosion and hydrolysis) by water absorption [24,25]. Furthermore, the shape of the inserted random PLGA sample changed to oval, which caused sample swelling. Both copolymers showed signs of recovery due to the elimination of angiogenesis (one of the processes contributing to inflammation) in the 3M and 4M samples. The tissues around the inserted random PLGA sample (Figure 11, Figure 12 and Figure 13(A-1 to A-6)) exhibited mild inflammation from the initial stage until 2 months. Additionally, inflammation by any macrophage, angiogenesis, or giant cell were not observed in the samples up to 3 months after the insertions [26]. Since the random PLGA samples were rapidly degraded and swelled, fewer stimuli caused by foreign-body reactions were reported [27,28].

On the other hand, the tissues around the inserted PGA–PLA block copolymer sample (Figure 11, Figure 12 and Figure 13(B-1 to B-6)) exhibited mild inflammation until 4 months after insertion. The PGA–PLA block copolymer showed slow degradation, slow swelling behavior, and fewer stimulated tissues, causing surface erosion, for 4 months (Figure 13(B-4)). This phenomenon was caused by the slow degradation of the surface erosion. Bulky hydrolysis was postponed in the following stage of degradation and the inserted sample’s rectangular shape was preserved for up to 6 months. Block copolymer, which is difficult to hydrolyze and residues with relatively high bulk molecular weights were the causes of the inhibition of the degradation. However, the random PLGA containing tissue after 6 months was not visible in the biopsy specimen because of its complete degradation (Figure 13(A-6)) [6].

## 4. Conclusions

Large-molecular-weight PGA–PLA block copolymers were synthesized in this study and, unlike the random PLGA, they demonstrated good biocompatibility and a slow rate of biodegradation. The random PLGA was found to be fully deteriorated after 3 months by the in vivo animal-degradation test (which was supported by the SEM and XRD measurements and the optical-analysis test), whereas the PGA–PLA block copolymer was still intact and thermally stable 6 months after insertion surgery. Furthermore, the histochemical analyses revealed that the PGA–PLA block copolymer did not exhibit any inflammation or irritation due to the breakdown by-product. Therefore, the PGA–PLA block copolymers were good alternatives to spinal-fixation material for long-term durability.

## Figures and Tables

**Table 1:** Characterization of PGA–PLA block copolymer.

Name	Tg (°C) ^a^		Tm (°C) ^a^		Molecular Weight ^b^ (M_w_)	Flexural Strength ^c^ (MPa)
	PLA	PGA	PLA	PGA		
PGA–PLA block copolymer	40.44	58.87	167.84	218.11	159,000	1*37*

^a^ DSC, 0~270 °C, heating rate: 10 °C/min, 2nd run, N_2_(g) condition; ^b^ GPC, 40 °C, Elute of HFIP: 0.3 mL/min, concentration: 3 mg/mL, PMMA standard; ^c^ UTM according to ISO 178:2019, flexion speed: 2 mm/min, span length: 64 mm, *n* = 5.

## Data Availability

Not applicable.

## Appendix A

From the in vivo animal-degradation test, the inserted specimens were extracted once a month up to half a year after implantation surgery, and the harvested specimens of the PGA–PLA block copolymer and random PLGA were characterized by thermogravimetric analysis equipment (TGA, TGA Q50, TA instruments Inc., New Castle, DE, USA) with a heating rate of 10 °C/min and a temperature scan ranging from 30 to 600 °C under a nitrogen atmosphere for the confirmation of biodegradation.

As shown in Figure A1, the temperature of the half-mass trace rapidly dropped in the random PLGA sample after 2-month insertion period. Due to the original shape loss, it was no longer measurable in the random PLGA sample. In the random PLGA, the rapid decrease in thermal stability and weight was based on bulk degradation. However, the temperature of the half-mass trace of the PGA–PLA block copolymer was maintained above 300 °C until the 6-month insertion period.
